# Patient perspectives of the influence of severe and non‐severe asthma on their quality of life: A national survey of asthma patients in Spain

**DOI:** 10.1111/crj.13461

**Published:** 2021-11-17

**Authors:** Eusebi Chiner, Carme Hernández, Marina Blanco‐Aparicio, Eunice Funenga‐Fitas, Carlos Jiménez‐Ruiz

**Affiliations:** ^1^ Pneumology Service Hospital Universitari Sant Joan d'Alacant Alicante Spain; ^2^ Home Hospitalization Unit, Medical and Nursing Direction, Hospital Clinic, Institut d'Investigacions Biomèdiques August Pi i Sunyer (IDIBAPS) Universitat de Barcelona Barcelona Spain; ^3^ Pneumology Service Hospital Universitario A Coruña Coruña Spain; ^4^ AstraZeneca Farmacéutica Spain S.A Madrid Spain; ^5^ Smoking Cessation Service Community of Madrid Madrid Spain

**Keywords:** asthma, burden of disease, disease management, quality of life

## Abstract

**Introduction:**

Little is known about adult asthma patients' perspective of their disease burden. This study aimed to obtain a comprehensive picture of patient needs, evaluate their knowledge, source of information, and perception of the severity of their asthma, and compare these variables between severe (SA) and non‐severe (NSA) asthma patients.

**Methods:**

We conducted an online cross‐sectional survey in Spain among asthma patients aged ≥18 years. A bespoke questionnaire was used to collect sociodemographic data, asthma characteristics, treatments, disease burden, patient's perception of disease severity, and asthma information sources. Patients were classified as SA and NSA according to GINA 2020 treatment steps recommendations. To compare populations, 600 participants (200 SA and 400 NSA) were randomly selected to complete the survey.

**Results:**

Participants were mostly women, mean age >38 years. SA patients underestimated the severity of their asthma; 52% judged it as mild, and only 2% considered their asthma severe. Overall, 50% of NSA and 96% of SA patients had experienced ≥1 exacerbation the previous year (*p* < 0.001). Fewer asthma exacerbations (SA) and improved quality of life (QoL) (NSA) were the most frequently expected therapy outcomes. NSA patients believe that asthma impacts their daily life (37%) and worsens QoL (34%) to a lesser degree than SA (67% and 59%, respectively; *p* < 0.001). Patient‐preferred sources of information were specialists (NSA:42%; SA: 38%) and primary care physicians (NSA: 41%; SA: 33%).

**Conclusions:**

Despite the effective therapies currently available, the results of this study still show a significant emotional burden and QoL impairment in patients with severe asthma.

## 1 INTRODUCTION

Asthma is a long‐term respiratory condition that can negatively impact on patients' health‐related quality of life (QoL) and carries a significant social and economic burden.^1^ Approximately 400 million people around the world and nearly 2 million people in Spain suffer from asthma, and prevalence has increased in recent decades, especially among the young.^2^

Severe asthma is defined as “asthma which requires treatment with high‐dose inhaled corticosteroids (ICS) plus a second controller (and/or systemic CS) to prevent it from becoming *uncontrolled* or which remains *uncontrolled* despite this therapy,” as specified by ERS/ATS guidelines definitions.^3^ According to the therapeutic steps of the Global Initiative for Asthma (GINA) 2020 report, severe asthma (SA) is defined as asthma that is uncontrolled despite adherence with maximal step 4 or step 5 therapy or that worsens when high‐dose treatment is decreased.^4^ Approximately 3–10% of people with asthma have severe refractory asthma.^5^ In Spain, 3.9% of asthmatic patients present severe uncontrolled asthma.^6^

Achieving and maintaining optimal asthma control is a major objective of asthma management. International and national guidelines stipulate goals for optimizing asthma management, such as preventing chronic symptoms, minimizing exacerbations and emergency care, minimizing the use of rescue short‐acting β2‐agonists (SABA), and maintaining regular physical activity.^4^, ^7^ Despite the periodic publication of these guidelines and the effective therapies currently available, the evidence indicates a lack of control among asthma patients,^8^ with a negative impact on their health status and on resource utilization and costs for the health system.^9^ Many patients may overestimate their symptom control and underestimate the severity of their condition, indicating that they tolerate symptoms and lifestyle limitations.^10^ A study from Spain reported that only 10% of patients with severe asthma have their condition controlled according to Spanish asthma guidelines (GEMA) criteria.^11^ Furthermore, patients and physicians overestimate asthma control, although overestimation by patients is greater. In fact, the CHAS study performed 10 years ago concluded that in Spain, asthma was still uncontrolled and defined the factors leading to this situation.^12^ A recent ethnographic study found that patients take years to understand and accept the chronic nature of their disease, delaying lifestyle adaptations.^13^

To the best of our knowledge, no survey has previously been conducted in a large sample of persons with asthma in Spain to understand patients' perceptions of the burden of disease according to severity. The aim of this survey was to draw a comprehensive picture of the needs of adult asthma patients in order to identify the factors that patients consider essential for the management of their condition, evaluate their level of knowledge, source of information and perceptions of the severity of their asthma, and compare these variables between severe and non‐severe asthma populations. We also aimed to identify unmet needs to optimize communication between patients and healthcare professionals.

## 2 MATERIALS AND METHODS

### 2.1 Study population

A cross‐sectional survey of patients ≥18 years with asthma was conducted between March 4 and March 17, 2020. In order to stratify the target population (patients with severe and non‐severe asthma), 84 976 individuals from the general population of the Ipsos Spain database were randomly invited by email to respond to an initial online questionnaire related to the diagnosis of asthma and treatment. The participants were asked about their diseases in an initial online questionnaire that included asthma as an option. Respondents who self‐reported having asthma were included in the subsequent analyses. Participants were asked to specify which of the following treatments they were receiving at the time of completing the questionnaire: injectables (biologics, such as omalizumab, mepolizumab, reslizumab, or benralizumab), oral therapies (leukotriene receptor antagonist or oral corticosteroids [OCS]), and inhaled therapies (long‐acting β2‐agonists [LABA], such as salmeterol; ICS plus LABA, such as fluticasone/salmeterol, budesonide/formoterol, beclomethasone/formoterol, or fluticasone/vilanterol; short‐acting β2‐agonists, such as salbutamol or terbutaline; and long‐acting muscarinic antagonists [LAMA], such as tiotropium bromide). According to their responses, participants were classified as having SA when they met one of the following criteria: (i) receiving biologics, (ii) receiving high‐dose ICS plus LABA plus LAMA, or (iii) receiving high‐dose ICS plus LABA, with or without a leukotriene receptor antagonist. All other patients were classified as NSA. After targeting populations, the main interview was conducted online using a semi‐structured self‐administered website questionnaire that required an average of 15 min to complete. Adult patients were eligible for the study if they were at least 18 years of age, self‐reported a previous diagnosis of asthma by a doctor, and had the ability to give informed consent. Patients in the Ipsos Spain database represent ~0.18% of the Spanish population, and geographical distribution of participants was representative of the Autonomous Communities.

### 2.2 Questionnaire

A bespoke questionnaire was developed for the study, which, while not formally validated, was created specifically to address the study objectives. All participants completed the questionnaire in Spanish. This tool consisted of 24 multiple‐choice questions that provided information on the following aspects: patient demographics, their relationship to other prevalent diseases (effect, fear, and knowledge), medical intervention for asthma in the preceding year, incidence of exacerbations (defined as acute episodes of progressively increasing shortness of breath, cough, wheezing, chest tightness, or some combination of these symptoms, both when these episodes required hospital admission/emergency visits, or when they were controlled by the patient by increasing their regular inhaled therapy), perception of the burden of symptoms and disease severity, social and emotional impact of asthma on everyday life, disease knowledge, preferred medication attributes, satisfaction with current therapies, and preferred source of information.

### 2.3 Statistical analyses

We followed the Strengthening the Reporting of Observational Studies in Epidemiology (STROBE) guidelines for reporting the study's findings.

Completed questionnaires were analyzed for the total population and by asthma severity. A sample size of 600 participants was required to obtain a margin of error of 4% and a 95% confidence interval. In order to increase statistical power for the comparative analyses, the participants were randomly chosen from each group to adjust the 1:2 ratio between SA and NSA (200 patients with SA, error ±6.93% and 400 patients with NSA, error ±4.90%).

Descriptive statistics appropriate for the measurement level (e.g., percentages for categorical variables; mean and standard deviation for continuous variables) were used. Independent Student *t* tests were used to compare the means of continuous variables, and chi‐square tests were used to test differences in proportions of categorical variables between groups. All analyses were performed in SPSS v. 25.0.32. A *p* value <0.05 was considered significant.

### 2.4 Ethical considerations

This study was conducted in accordance with the confidentiality guidelines set down in the International Chamber of Commerce/European Society for Opinion and Marketing Research (ICC/ESOMAR) and European Pharmaceutical Market Research Association (EphMRA) codes of conduct and conformed to the requirements of Spanish legislation in terms of confidentiality and data protection.

### 2.5 Data availability

The data that support the findings of this study are available from the corresponding author on reasonable request.

## 3 RESULTS

A total of 10 000 respondents completed the initial questionnaire. Of these, 862 (8.6%) were subjects with self‐reported, physician‐diagnosed asthma; 350 (3.1%) were categorized as SA (GINA Steps 4–5), while 512 (5.1%) were categorized as NSA (GINA Steps 1–3). Six hundred participants (200 with SA and 400 with NSA) randomly selected completed the full interview. Baseline demographic and clinical characteristics of participants according to asthma severity are presented in Table 1. Participants in both groups were predominantly women (NSA: 61.5%, SA: 67.0%); mean age was over 38 years, and the age range was evenly matched between groups. Most participants were employed (NSA: 78.8%; SA: 84.0%) and had completed secondary education. Compared with NSA, higher education was significantly more frequent in the SA group (51.8% vs. 60.5%, *p* = 0.042). According to the comorbidities that participants were asked if they had at the time of the study, diabetes (NSA: 7.2%; SA:19.5%) and mental disorders (NSA: 4.2%; SA: 13.0%) were the most common. All comorbidities were significantly more prevalent in participants with SA (*p* < 0.05), except stroke and degenerative diseases, for which no significant differences were found. A diagnosis of asthma and chronic obstructive pulmonary disease (COPD) was reported by 1.2% of NSA and 8.0% of SA patients (*p* < 0.001). Asthma medications taken at the time of the survey are summarized in Table 1. Overall, 25.0% of NSA and 36.5% of SA patients said they had received at least one prescription of OCS within the previous 12 months (*p* = 0.003). Significantly more SABA was prescribed in the NSA group (87.5% vs 74.0%; *p* < 0.001). In contrast, 18.8% of NSA and 80.0% of SA patients were prescribed ICS/LABA (*p* < 0.001).

**TABLE 1:** Demographics and clinical characteristics of participants

	Non‐severe asthma	Severe asthma	*p* value
Subjects, *n*	400	200	
Females, *n* (%)	246 (61.5)	134 (67.0)	0.188
Age (years), mean (SD)	38.49 (10.31)	38.96 (10.29)	0.598
Age group, *n* (%)			0.979
18–24	43 (10.8)	21 (10.5)	
25–34	99 (24.8)	46 (23)	
35–44	133 (33.2)	70 (35)	
45–54	102 (25.5)	50 (25)	
≥55	23 (5.8)	13 (6.5)	
Married	168 (42.0)	111 (55.5)	***0.001***
Place of residence, *n* (%)			
Barcelona	28 (7.0)	14 (7.0)	1.000
Canary Islands	12 (3.0)	9 (4.5)	0.345
Center	28 (7.0)	14 (7.0)	1.000
Levante	50 (12.5)	25 (12.5)	1.000
Madrid	86 (21.5)	40 (20.0)	0.617
North	43 (10.8)	23 (11.5)	0.782
Northeast	30 (7.5)	11 (5.5)	0.630
Northwest	39 (9.8)	21 (10.5)	0.772
South	84 (21.0)	43 (21.5)	0.887
Educational level, *n* (%)			
Primary education	5 (1.2)	2 (1.0)	1.000
General Certificate of Secondary Education	30 (7.5)	10 (5.0)	0.247
Vocational Education and Training	28 (7.0)	10 (5.0)	0.343
General Certificate of Education	57 (14.2)	16 (8.0)	***0.027***
Certificate of Higher Education	73 (18.2)	41 (20.5)	0.508
University degree/Further education	207 (51.8)	121 (60.5)	***0.042***
Employed, *n* (%)	315 (78.8)	168 (84.0)	0.126
Comorbidities *n* (%)^a^			
Diabetes	29 (7.2)	39 (19.5)	***<0.001***
Mental disorders	17 (4.2)	26 (13.0)	***<0.001***
COPD	5 (1.2)	16 (8.0)	***<0.001***
Ischemic heart disease	2 (0.5)	8 (4.0)	***0.003***
Stroke	4 (1.0)	6 (3.0)	0.092
Cancer	2 (0.5)	7 (3.5)	***0.008***
Degenerative diseases	3 (0.8)	5 (2.5)	0.125
AIDS	1 (0.2)	5 (2.5)	***0.017***
Number patients with OCS prescription last year, *n* (%)	100 (25.0)	73 (36.5)	***0.003***
Number of OCS prescription last year, *n* (%)^b^			
1–3	66 (38.2)	38 (22.0)	0.064
4–6	23 (13.3)	21 (12.1)	0.390
≥7	11 (11.0)	14 (19.2)	0.131
Mean OCS prescription last year (SD)	5.3 (10.1)	7.5 (14.5)	***0.031***
Non‐OCS asthma treatment currently received, *n* (%)			
SABA	350 (87.5)	148 (74.0)	***<0.001***
LABA	5 (1.3)	4 (2.0)	0.498
ICS/LABA	75 (18.8)	160 (80.0)	***<0.001***
LAMA + inhaler	7 (1.8)	50 (25.0)	***<0.001***
Any LTRA	66 (16.5)	89 (44.5)	***<0.001***
Any biologics	0 (0.0)	66 (33.0)	***<0.001***

Abbreviations: COPD, chronic obstructive pulmonary disease; ICS, inhaled corticosteroids; LABA, long‐acting β2‐agonists; LAMA, long‐acting muscarinic antagonists; LTRA, leukotriene receptor antagonists; SABA, short‐acting β2‐agonists; SD, standard deviation.

^a^ Participants were asked which of the listed diseases they were currently suffering from at the time of the survey.

^b^ Frequency is relative to the participant with OCS prescriptions.

### 3.1 Asthma perceptions, exacerbations, and hospitalizations

**FIGURE 1:**
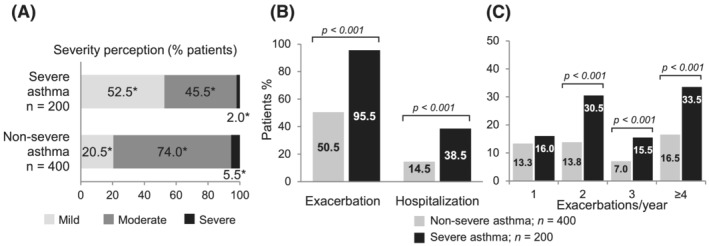
(A) Severity perception by participant according to GINA steps (Mild: GINA steps 1–2; Moderate: GINA step 3; Severe: GINA steps 4–5). (B) Frequency of exacerbation and hospitalization among participants in the last year; (C) number of exacerbations in the 12 months before the survey. *p < 0.05

Severity perception and incidence of exacerbations and hospitalizations are shown in Table 2. The general perception of asthma severity was more aligned to asthma classification in the NSA group, with 95% of the NSA respondents reporting their asthma as mild to moderate. In contrast, concordance between perceived and real severity among SA respondents was only 2% (Figure 1A). The majority of patients with SA reported ≥1 exacerbation within the previous 12 months (95.5%) compared with NSA subjects (50.5%; *p* < 0.001), and the percentage of patients who reported ≥4 exacerbations in the last year was significantly higher for SA patients compared to those with NSA (33.5% vs 16.5%; *p* < 0.001). Hospitalizations were also significantly more frequent in the SA group (≥1 hospitalization: 38.5% for SA vs. 14.5% for NSA; *p* < 0.001), and these patients also received systemic steroids during hospitalization more frequently than NSA patients (mean [SD]: 2.31 [2.67] times vs. 1.53 [0.82] times; *p* = 0.033) (Figure 1B,C).

**TABLE 2:** Perceptions of asthma and disease characteristics by asthma severity

	Non‐severe asthma	Severe asthma	*p* value
Subjects *n*	400	200	
Patients' severity perception, *n* (%)			
Mild	41 (20.5)	210 (52.5)	***<0.001***
Moderate	148 (74.0)	182 (45.5)	***<0.001***
Severe	11 (5.5)	8 (2.0)	***0.021***
Number of patients with exacerbation last year, *n* (%)	**202 (50.5)**	**191 (95.5)**	***<0.001***
Mean of exacerbations last year (SD)	**3.86 (7.4)**	**4.57 (8.8)**	0.302
Number of exacerbations during last year, *n* (%)			
0	198 (49.5)	9 (4.5)	***<0.001***
1	53 (13.3)	32 (16.0)	0.362
2	55 (13.8)	61 (30.5)	***<0.001***
3	28 (7.0)	31 (15.5)	***<0.001***
≥4	66 (16.5)	67 (33.5)	***<0.001***
Number of patients hospitalized in the last year, *n* (%)	**58 (14.5)**	**77 (38.5)**	***<0.001***
Mean of hospitalizations in the last year (SD)	**1.38 (0.52)**	**1.95 (1.33)**	***<0.001***
Number of hospitalizations during last year, *n* (%)			
0	342 (85.5)	123 (61.5)	***<0.001***
1	37 (9.3)	39 (19.5)	***<0.001***
2	20 (5.0)	22 (11.0)	***0.007***
≥3	1 (0.3)	16 (8.0)	***<0.001***
Mean SCS (OCS or IvCS) treatment during hospitalization (SD)	**1.53 (0.82)**	**2.31 (2.67)**	***0.033***

Abbreviations: IvCS, intravenous corticosteroid; OCS, oral corticosteroid; SCS, systemic corticosteroid.

### 3.2 Treatment experience and expectation

**FIGURE 2:**
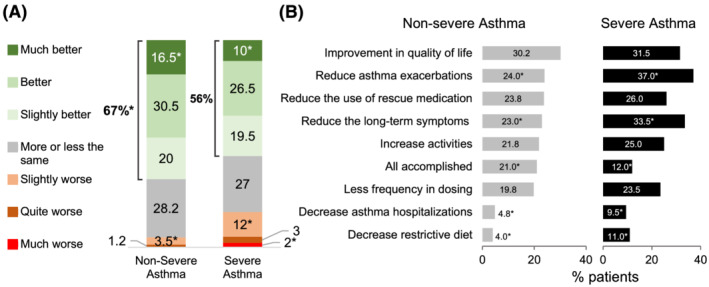
Current treatment experience (A) and expectations for improvement (B) at the time of the survey. *p < 0.05

Perceived treatment expectations and improvements at the time of the survey are shown in Figure 2. Overall, most participants felt better about their current asthma treatment (after being asked “how do you feel about your current treatment as compared with the expectations you had before initiating it”), and this perception was more pronounced in NSA than in SA patients (67% vs. 56%; *p* = 0.008). When participants were asked about *what improvements they expected from their current treatment*, the most frequently mentioned options were to improve QoL (32%) for the NSA group and to reduce asthma exacerbations (37%) for the SA group. Notably, 21% of NSA and 12% of SA patients responded that all expectations had been met (additional data in Table S1).

### 3.3 Attitude towards asthma, quality of life, and work productivity

**FIGURE 3:**
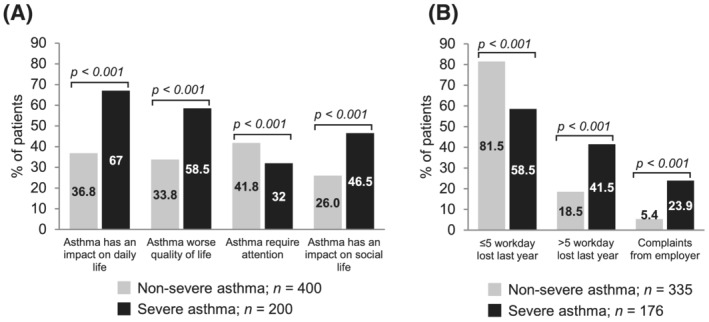
Perception of the impact of asthma on quality of life (A) and work productivity (B)

Overall, most participants said they had a proactive attitude towards their disease and felt they had good knowledge about their asthma but worried about it. SA patients affirmed that their asthma had an impact on their daily life (67%) and worsened their QoL (58.5%) significantly more frequently than NSA patients (36.8% and 33.8%; *p* < 0.001) (Figure 3A). There were significant differences in the emotional state related to asthma in the groups: NSA patients felt anger about their asthma (20%) and SA patients felt fatigued and concerned about their asthma (20%) (additional data in Tables S1 and S2).

Absenteeism due to asthma was more pronounced in SA patients, who reported having more frequently missed >5 workdays in the last year than NSA patients (41.8% vs. 18.5%; *p* < 0.001) (Figure 3B). Notably, 23.9% of SA patients had received complaints from employers compared to 5% of NSA patients (*p* < 0.001).

**FIGURE 4:**
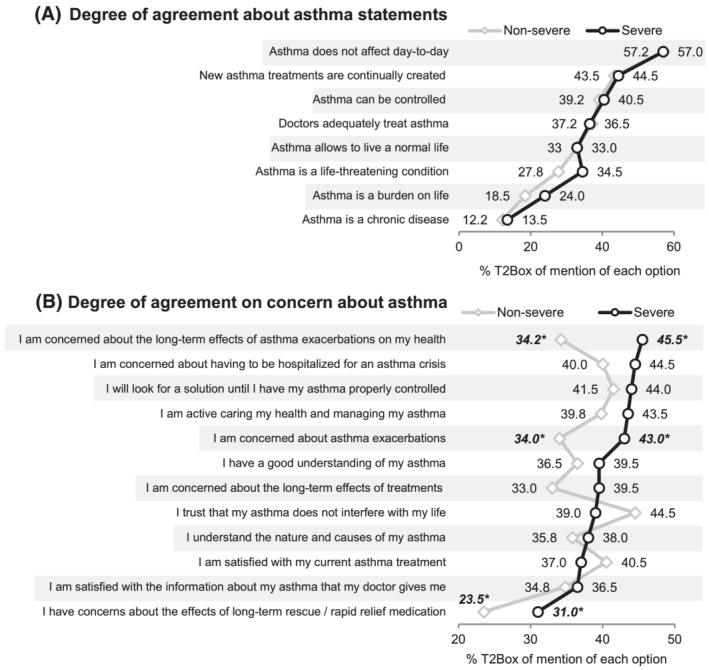
Degree of agreement on statement (A) and concerns (B) about asthma. Top 2 box (%T2Box) denotes the proportions of patients given the two highest possible degrees of the agreement for each sentence (1 = do not agree and 7 = strongly agree). *p < 0.05

Figure 4 shows the level of agreement on several asthma‐related statements (Figure 4A) and concerns (Figure 4B) in both severity groups. Asthma chronicity is the statement that generates the greatest consensus in both groups (Figure 4A). Also, while SA patients are more concerned about asthma, NSA patients are more confident that the disease does not interfere with their lives. Moreover, only 27.8% of NSA and 34.5% of SA participants affirm that asthma can be controlled and 12.2% of NSA and 13.5% of participants believe that asthma does not affect their daily life (additional data in Table S3).

### 3.4 Perceptions of common diseases

**FIGURE 5:**
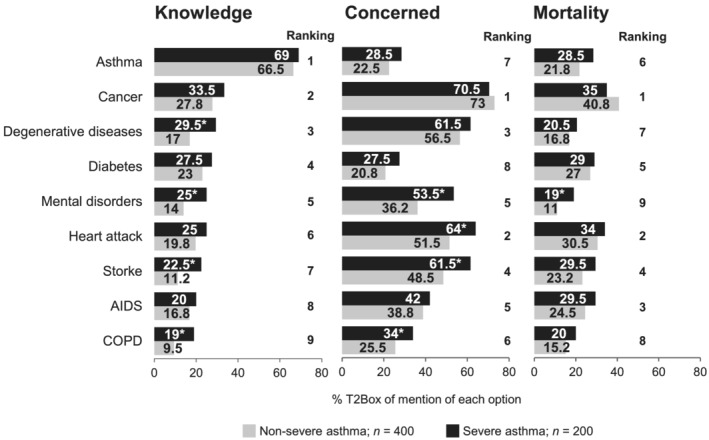
Attitudes, concerns, and mortality perception about common disease reported by participants. Top 2 box (%T2Box) denotes the proportions of patients giving the two highest possible degrees of agreement for each sentence. *p < 0.05

Perception and knowledge of other diseases are represented in Figure 5. Generally, the perception of participants was that they had good knowledge about asthma, but their reported knowledge of other diseases was lower. However, asthma generated low concern (seventh in the ranking of nine diseases) and was considered a lower risk of mortality (sixth in the ranking of nine diseases). Cancer and heart attack were selected as more worrisome diseases and were considered the deadliest diseases by all participants.

### 3.5 Sources of asthma information

**FIGURE 6:**
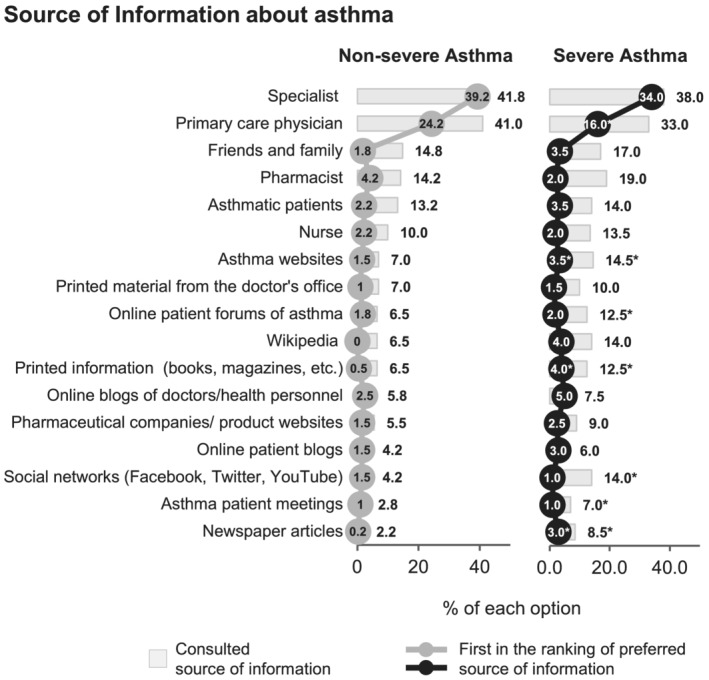
Sources of information consulted about asthma (bars) and % of the first options in the ranking of preferred source of information (lines). *p < 0.05

Primary care physicians and specialists were the main sources of information about asthma for NSA (82.8%) and SA (71%) patients (Figure 6), and the specialist was the preferred source of information of all participants. Online information was consulted more often by SA than NSA patients: specific disease‐ or health‐related websites (14.7% vs. 7.0%; *p* = 0.003); online patient forums of asthma (12.5% vs. 6.5%; *p* = 0.013); Wikipedia (14.0% vs. 6.5%; *p* = 0.002); and social networks (Facebook, Twitter, and YouTube) (14.0% vs. 4.2%; *p* < 0.001). Notably, 22.5% of NSA and 16.0% of SA patients said that they did not seek additional information on their condition and treatments beyond that received during consultations (additional data in Table S4).

## 4 DISCUSSION

This study explores perceptions surrounding asthma and its impact on QoL in a Spanish cohort of asthma patients with severe and non‐severe disease. According to this study, the perception of asthma severity was underestimated by 95% of the SA patients, a result consistent with previous reports.^11^, ^14^ This may be related to a poor understanding of disease severity by patients and healthcare professionals. Given that the assessment of asthma severity determines the medications required to achieve disease control, it is reasonable to think that patients with a misperception of asthma severity might not achieve optimal control. Indeed, in the 12 months preceding the survey, SA patients reported a significant number of exacerbations and hospitalizations. This is a striking observation in patients whose asthma was considered non‐severe at the time of the survey, and could reflect poor asthma control, since underestimation of asthma severity and a low concordance rate between patients and professionals may contribute to poor treatment adherence, and as a result, poor asthma control.^15^ Large asthma surveys conducted in America, the Asia‐Pacific region and Europe have also shown that significantly underestimated severity is associated with undertreatment of asthma, suggesting that improvements are needed in long‐term asthma management.^16^, ^17^, ^18^ It is now agreed that asthma control can be improved when patient perspectives are considered.^19^ Even though all participants reported having a high level of knowledge of asthma compared to other common diseases, it is the disease they are least afraid of (second only to diabetes) and they do not consider it life‐threatening. This may explain their low perceived severity of the disease.

We found that SA patients received more OCS prescriptions and had more exacerbations than NSA patients. This was to be expected considering the GINA 2020 recommendation for prescribing OCS in patients with severe, uncontrolled asthma.^4^ However, the latest treatment recommendations do not include maintenance OCS as the preferred treatment, underlining the need for educating general practitioners on current guidelines and issues associated with OCS use. Moreover, the recent introduction of biological OCS‐sparing therapies showing relevant benefits^20^ was reflected by a proportion of 33% of SA patients who received biologics. Treatment with OCS in some patients with SA improves asthma control and reduces exacerbation rates, but there is strong evidence to support the proposal that patients with SA have substantial excess morbidity from multiple diseases and adverse effects associated with OCS exposure.^21^, ^22^ In fact, comorbidities associated with OCS were substantially more frequent in SA patients. Most participants were prescribed SABA, but ICS/LABA was more frequently prescribed in the SA group, which would indicate undertreatment and poor asthma control in the NSA group. In the last few years, evidence on the risks associated with high SABA use has grown substantially.^23^, ^24^ In this line, a recent European study reported a 29% rate of SABA overuse in Spain.^25^

Although both groups had a positive attitude to and extensive knowledge of asthma, significantly more patients with SA were concerned about their disease and recognized that asthma has a detrimental effect on their daily life, social integration, and overall QoL. In both groups, patient expectations of asthma management were modest; few participants believed that asthma could be controlled and did not affect their day‐to‐day lives. In addition, SA patients were more worried about the long‐term effects of asthma exacerbations, and they expected that medication would improve this aspect. These attitudes can have a negative impact on patient care and QoL since asthma management practices and the knowledge, attitudes, and behavior of adult asthma patients in the general population are associated with their degree of asthma control.^26^ Asthma also imposes a high social burden in terms of loss of work productivity. Remarkably, SA patients have received complaints from employers due to workdays lost attributable to asthma, which suggests that a great effort is still needed to improve levels of social awareness about chronic respiratory conditions such as asthma. A European study showed the negative emotional impact of uncontrolled asthma on employees in the workplace and their productivity while at work.^27^ In this line, a Canadian study with 300 patients with asthma showed that over one‐third of patients with asthma had psychological distress (depressive and anxiety symptomatology) as a comorbidity, and this affected both absenteeism and presenteeism.^28^

Most participants indicated that they preferred the information that came from healthcare professionals, emphasizing the need for both patients and professionals to have the same perception of asthma severity and control. It has been well documented that both patients and physicians overestimate control, whereas overestimation by patients is greater, particularly in uncontrolled asthma.^11^, ^14^, ^29^ This poor concordance should be addressed in education programs, mainly for patients with severe, uncontrolled asthma. It is worth noting that nearly 20% of participants did not request information about the management of their condition. If this perception is true, the opportunity for misinterpreting disease control is significant.

The strengths of this study include a large sample size and a study design that enables the evaluation of a large sample of patients stratified by asthma severity that were representative of asthma patients in Spain, and similar to a population reported in another comparable study.^30^

This study has some limitations. First, survey data were generated by patient self‐assessment and, as such, must be viewed in the context of representing the perceptions of patients, and the outcomes have not been clinically verified. In addition, The GINA 2020 step was used as a proxy to assess asthma severity without considering symptoms and clinical implications, as these may residual confounding elements that can lead to classification bias. As a result, the severity level of some patients may not be adequately defined. The GINA 2020 step was used as a proxy to assess asthma severity without considering symptoms and clinical implications may have resulted in classification bias, therefore constituting residual confounding. An implication of this strategy may be that some patients' severity level was not adequately defined.

Another limitation is that it was a cross‐sectional survey and therefore, cause and effect relationships cannot be established. Furthermore, we used a non‐validated questionnaire to assess the impact of the disease and satisfaction with the treatment, while we could have used a validated questionnaire to evaluate disease control. However, none of the validated questionnaire could fully meet all the study objectives.

In summary, despite the effective therapies currently available, our results show that asthma still has a significant emotional burden and impairs the QoL of patients with this disease. This is especially true of patients with severe asthma. The impact on daily life, workdays lost, and a considerable number of exacerbations and hospitalizations are indicators that there is still room for a massive improvement in the management of asthma. The survey also gathered a large amount of novel information on patients' attitudes toward asthma. This could be used to analyze patients' behavior and attitudes to improve various sources of information about asthma, such as websites and patient support organizations, that could effectively complement and increase knowledge and skills to help patients collaborate effectively with healthcare professionals in the management of their disease.

## CONFLICT OF INTEREST

Eunice Fitas is an employee of AstraZeneca Farmacéutica Spain S.A.

## AUTHOR CONTRIBUTIONS

All authors contributed equally to this manuscript and were responsible for data analysis and interpretation. All authors drafted the manuscript, provided critical revisions, and approved the final version of the manuscript for submission.

## ETHICS STATEMENT

The inclusion of cases was carried out in accordance with the requirements expressed in the Declaration of Helsinki (revision of Tokyo, October 2004) and the Spanish Organic Law on Data Protection 15/1999. The study was approved at San Juan de Alicante University Hospital Ethics Committee.

## Supporting information


**Table S1.** Treatment experience and expectation
**Table S2.** Attitude towards asthma, quality of life, and work productivity impact
**Table S3**. Degree of agreement: definitions, uses and attitudes towards asthma
**Table S4.** Source of information consulted and ranking of preferred source of information

## Data Availability

The data that support the findings of this study are available from the corresponding author on reasonable request.
