# Supplementary material for: Patient perspectives of the influence of severe and non‐severe asthma on their quality of life: A national survey of asthma patients in Spain
Source: Clin Respir J. 2021 Nov 17;16(2):130–41. doi: 10.1111/crj.13461 (PMC9060022; doi:10.1111/crj.13461)
Supplement: Supplementary file 1 — Table S1. Treatment experience and expectation Table S2. Attitude towards asthma, quality of life, and work productivity impact Table S3. Degree of agreement: definitions, uses and attitudes towards asthma Table S4. Source of information consulted and ranking of preferred source of information [file CRJ-16-130-s001.docx]

Appendix A. Supplementary Tables

| **Table S1.** Treatment experience and expectation | | | |
| --- | --- | --- | --- |
|  | **Non-severe asthma** | **Severe asthma** | ***p-*value** |
| **Subjects *n*** | 400 | 200 |  |
| **With regard to your current treatment, how do you feel about the expectations you had before you started it? *n* (%)** |  |  |  |
| Much worse | 0 (0.0) | 4 (2.0) | ***0.012*** |
| Quite worse | 5 (1.2) | 6 (3.0) | 0.132 |
| Slightly worse | 14 (3.5) | 24 (12.0) | ***< 0.001*** |
| More or less the same | 113 (28.2) | 54 (27.0) | 0.747 |
| Slightly better | 80 (20.0) | 39 (19.5) | 0.885 |
| Quite better | 122 (30.5) | 53 (26.5) | 0.310 |
| Much Better | 66 (16.5) | 20 (10.0) | ***0.032*** |
| *All better* | 268 (67.0) | 112 (56.0) | ***0.008*** |
| *All worse* | 19 (4.8) | 34 (17.0) | ***< 0.001*** |
| **What improvements do you expect from your current treatment? *n* (%)** |  |  |  |
| Improved quality of life | 121 (30.2) | 63 (31.5) | 0.754 |
| Reduced asthma exacerbations | 96 (24.0) | 74 (37.0) | ***< 0.001*** |
| Reduced the use of rescue medication | 95 (23.8) | 52 (26.0) | 0.546 |
| Reduced long-term symptoms | 92 (23.0) | 67 (33.5) | ***0.006*** |
| Increased activities | 87 (21.8) | 50 (25.0) | 0.371 |
| All expectations met | 84 (21.0) | 24 (12.0) | ***0.007*** |
| Less frequency in dosing | 79 (19.8) | 47 (23.5) | 0.288 |
| Fewer asthma hospitalizations | 19 (4.8) | 19 (9.5) | ***0.024*** |
| Less restrictive diet | 16 (4.0) | 22 (11.0) | ***< 0.001*** |

| **Table S2.** Attitude towards asthma, quality of life, and work productivity impact | | | |
| --- | --- | --- | --- |
|  | **Non-severe asthma** | **Severe asthma** | ***p-*value** |
| **Subjects *n* (%)** | 400 | 200 |  |
| **Attitude towards asthma, *n* (%)** |  |  |  |
| Active attitude towards the disease | 270 (67.5) | 134 (67.0) | 0.902 |
| Good knowledge of the disease | 247 (61.8) | 128 (64.0) | 0.592 |
| Worried about asthma | 239 (59.8) | 135 (67.5) | 0.065 |
| General negative attitude towards the disease | 216 (54.0) | 91 (45.5) | 0.05 |
| Asthma has an impact on daily life | 147 (36.8) | 134 (67.0) | ***< 0.001*** |
| Asthma worsens quality of life | 135 (33.8) | 117 (58.5) | ***< 0.001*** |
| Asthma requires attention | 167 (41.8) | 64 (32.0) | ***< 0.001*** |
| Satisfied with current treatment but doubts for the long term | 147 (36.8) | 78 (39.0) | 0.592 |
| Asthma has an impact on social life | 104 (26.0) | 93 (46.5) | ***< 0.001*** |
| Satisfied with treatment in long and short term | 135 (33.8) | 53 (26.5) | 0.071 |
| Unsatisfied with current treatment | 118 (29.5) | 69 (34.5) | 0.212 |
| **Regarding my asthma, my feelings are, *n* (%)** |  |  |  |
| anger | 80 (20.0) | 35 (17.5) | 0.463 |
| normal | 78 (19.5) | 20 (10.0) | ***0.003*** |
| serenity | 68 (17.0) | 22 (11.0) | 0.052 |
| fatigue | 65 (16.2) | 40 (20.0) | 0.254 |
| concern | 62 (15.5) | 40 (20.0) | 0.166 |
| confidence | 21 (5.2) | 9 (4.5) | 0.691 |
| sadness | 13 (3.2) | 17 (8.5) | ***0.005*** |
| loneliness | 8 (2.0) | 10 (5.0) | ***0.042*** |
| expectancy | 5 (1.2) | 7 (3.5) | 0.060 |
| **Workdays lost last year due to asthma; *n* (%)** |  |  |  |
| ≤5 days | 273 (81.5) | 103 (58.5) | ***< 0.001*** |
| > 5 days | 62 (18.5) | 73 (41.5) | ***< 0.001*** |
| **Complaints from employer** | 18 (5.4) | 42 (23.9) | ***< 0.001*** |

| **Table S3**. Degree of agreement: definitions, uses and attitudes towards asthma | | | |
| --- | --- | --- | --- |
|  | **Non-severe asthma** | **Severe asthma** | ***p-*value** |
| **Subjects *n*** | 400 | 200 |  |
| **Degree of agreement on asthma definitions, n (%) of T2B** |  |  |  |
| Asthma is a chronic disease | 229 (57.2) | 114 (57) | 0.953 |
| Asthma is a burden on life | 174 (43.5) | 89 (44.5) | 0.816 |
| Asthma is a life-threatening condition | 157 (39.2) | 81 (40.5) | 0.768 |
| Asthma allows me to live a normal life | 149 (37.2) | 73 (36.5) | 0.858 |
| Doctors adequately treat asthma | 132 (33) | 66 (33) | 1.000 |
| Asthma can be controlled | 111 (27.8) | 69 (34.5) | 0.089 |
| New asthma treatments are continually created | 74 (18.5) | 48 (24) | 0.115 |
| Asthma does not affect day-to-day life | 49 (12.2) | 27 (13.5) | 0.664 |
| **Degree of agreement uses and attitudes towards asthma; *n* (%) of T2B** |  |  |  |
| I am concerned about the long-term effects of asthma exacerbations on my health | 137 (34.2) | 91 (45.5) | **0.007** |
| I am concerned about having to be hospitalized for an asthma exacerbation | 160 (40.0) | 89 (44.5) | 0.292 |
| I will look for a solution until I have my asthma properly controlled | 166 (41.5) | 88 (44.0) | 0.559 |
| I am active in the care of my health and managing my asthma | 159 (39.8) | 87 (43.5) | 0.379 |
| I am concerned about asthma exacerbations | 136 (34.0) | 86 (43.0) | ***0.031*** |
| I have a good understanding of my asthma | 146 (36.5) | 79 (39.5) | 0.474 |
| I am concerned about the long-term effects of treatments | 132 (33.0) | 79 (39.5) | 0.116 |
| I trust that my asthma does not interfere with my life | 178 (44.5) | 78 (39.0) | 0.199 |
| I understand the nature and causes of my asthma | 143 (35.8) | 76 (38.0) | 0.589 |
| I am satisfied with my current asthma treatment | 162 (40.5) | 74 (37.0) | 0.408 |
| I am satisfied with the information about my asthma that my doctor gives me | 139 (34.8) | 73 (36.5) | 0.672 |
| I have concerns about the effects of long-term rescue/rapid relief medication | 94 (23.5) | 62 (31.0) | ***0.048*** |

T2B: Top 2 Box

| **Table S4.** Source of information consulted and ranking of preferred source of information | | | | | | | |
| --- | --- | --- | --- | --- | --- | --- | --- |
|  | *Consulted* | | |  | *First in ranking ^a^* | | |
|  | **Non-severe asthma** | **Severe asthma** | ***p-*value** |  | **Non-severe asthma** | **Severe asthma** | ***p*-value** |
| **Subjects; *n*** | 400 | 200 |  |  | 400 | 200 |  |
| **Source of information, *n* (%)** |  |  |  |  |  |  |  |
| Specialist | 167 (41.8) | 76 (38.0) | 0.378 |  | 157 (39.2) | 68 (34.0) | 0.210 |
| Primary care physician | 164 (41.0) | 66 (33.0) | 0.057 |  | 97 (24.2) | 32 (16.0) | ***0.020*** |
| Friends and family | 59 (14.8) | 34 (17.0) | 0.473 |  | 7 (1.8) | 7 (3.5) | 0.181 |
| Pharmacist | 57 (14.2) | 38 (19.0) | 0.133 |  | 17 (4.2) | 4 (2.0) | 0.238 |
| Asthma patients | 53 (13.2) | 28 (14.0) | 0.800 |  | 9 (2.2) | 7 (3.5) | 0.370 |
| Nurse | 40 (10.0) | 27 (13.5) | 0.199 |  | 9 (2.2) | 4 (2.0) | 1.000 |
| Asthma websites | 28 (7.0) | 29 (14.5) | ***0.003*** |  | 6 (1.5) | 7 (3.5) | 0.113 |
| Printed material from the doctor's office | 28 (7.0) | 20 (10.0) | 0.202 |  | 4 (1.0) | 3 (1.5) | 0.691 |
| Online asthma patient forums | 26 (6.5) | 25 (12.5) | ***0.013*** |  | 7 (1.8) | 4 (2.0) | 1.000 |
| Wikipedia | 26 (6.5) | 28 (14.0) | ***0.002*** |  | 0 (0.0) | 8 (4.0) | ***< 0.001*** |
| Printed information (books, magazines, etc.) | 26 (6.5) | 25 (12.5) | ***0.013*** |  | 2 (0.5) | 8 (4.0) | ***0.003*** |
| Online blogs by doctors/health personnel | 23 (5.8) | 15 (7.5) | 0.407 |  | 10 (2.5) | 10 (5.0) | 0.108 |
| Pharmaceutical companies/product websites | 22 (5.5) | 18 (9.0) | 0.105 |  | 6 (1.5) | 5 (2.5) | 0.389 |
| Online patient blogs | 17 (4.2) | 12 (6.0) | 0.346 |  | 6 (1.5) | 6 (3.0) | 0.216 |
| Social networks (Facebook, Twitter, YouTube) | 17 (4.2) | 28 (14.0) | ***< 0.001*** |  | 6 (1.5) | 2 (1.0) | 0.725 |
| Asthma patient meetings | 11 (2.8) | 14 (7.0) | ***0.014*** |  | 4 (1.0) | 2 (1.0) | 1.000 |
| Newspaper articles | 9 (2.2) | 17 (8.5) | ***< 0.001*** |  | 1 (0.2) | 6 (3.0) | ***0.007*** |
| Does not look for information | 90 (22.5) | 32 (16.0) | 0.062 |  | *NA* | *NA* | *NA* |

NA: Not applicable
